# Supplementary material for: MetaRibo-Seq measures translation in microbiomes
Source: Nat Commun. 2020 Jun 29;11:3268. doi: 10.1038/s41467-020-17081-z (PMC7324362; doi:10.1038/s41467-020-17081-z)
Supplement: Supplementary file 10 — Supplementary Data 7 [file 41467_2020_17081_MOESM10_ESM.zip › File2/Confidence_High_Taxonomy/123490_out.krona.html]

Javascript must be enabled to view this page.

members
magnitude
magnitudeUnassigned
count
unassigned
taxon
rank

123490\_out

126

126
superkingdom
2

976
phylum
1

class
1
200643

171549
order
1

171550
family
1

239759
genus
1


SRS011134\_contig\_number\_44126
1
species
1118061

1239
phylum
125

186801
class
124

124
order
186802

186803
123
3
family

SRS016267\_contig\_number\_contig-100\_29934.29934SRS018575\_contig\_number\_contig-100\_12112.12112SRS064645\_contig\_number\_contig-100\_18592.18592

297314

SRS024435\_contig\_number\_contig-100\_35787.117771
1
species

841
118
16
genus

SRS011302\_contig\_number\_contig-100\_2775.170073SRS011405\_contig\_number\_contig-100\_23503.23503SRS014923\_contig\_number\_13433SRS053398\_contig\_number\_6158SRS058070\_contig\_number\_24061SRS063985\_contig\_number\_contig-100\_21510.21510SRS074670\_contig\_number\_contig-100\_11839.11839SRS075078\_contig\_number\_contig-100\_6611.181094SRS077127\_contig\_number\_contig-100\_29206.71716SRS097958\_contig\_number\_contig-100\_24315.62471SRS1041037\_contig\_number\_contig-100\_7275.68136SRS1055056\_contig\_number\_9986SRS140513\_contig\_number\_contig-100\_34940.34940SRS143466\_contig\_number\_29877SRS147377\_contig\_number\_contig-100\_6715.48168SRS148159\_contig\_number\_31065

1262941
4
species

SRS015065\_contig\_number\_13945SRS077194\_contig\_number\_31883SRS100021\_contig\_number\_9946SRS147271\_contig\_number\_contig-100\_29.145969


SRS149879\_contig\_number\_25168
species
1
166486


SRS104912\_contig\_number\_10742SRS143780\_contig\_number\_4754
species
2
512314


SRS012273\_contig\_number\_23749SRS012969\_contig\_number\_30491SRS014313\_contig\_number\_5667SRS016989\_contig\_number\_11765SRS021948\_contig\_number\_contig-100\_1341.192598SRS024132\_contig\_number\_29604SRS024492\_contig\_number\_contig-100\_32319.32320SRS064557\_contig\_number\_19035SRS077231\_contig\_number\_contig-100\_90.81570SRS077454\_contig\_number\_contig-100\_18925.59121SRS077849\_contig\_number\_6344SRS144135\_contig\_number\_32665SRS144297\_contig\_number\_12592
13
species
1897017

12
species

SRS015431\_contig\_number\_52426SRS019068\_contig\_number\_32396SRS023526\_contig\_number\_38197SRS046712\_contig\_number\_1994SRS054956\_contig\_number\_10312SRS063040\_contig\_number\_8399SRS075878\_contig\_number\_14125SRS1041136\_contig\_number\_23491SRS1055022\_contig\_number\_24554SRS1055076\_contig\_number\_8679SRS146812\_contig\_number\_57063SRS147919\_contig\_number\_4510
301302


SRS051031\_contig\_number\_23306SRS097920\_contig\_number\_22789SRS1041144\_contig\_number\_contig-100\_4757.53416SRS140492\_contig\_number\_29309
species
4
360807

2293144
species
14

SRS015663\_contig\_number\_29174SRS017433\_contig\_number\_16809SRS018817\_contig\_number\_2804SRS042628\_contig\_number\_11209SRS048060\_contig\_number\_contig-100\_2740.42769SRS050422\_contig\_number\_33894SRS063127\_contig\_number\_379SRS076804\_contig\_number\_19270SRS077335\_contig\_number\_contig-100\_180.59885SRS077641\_contig\_number\_contig-100\_5362.101878SRS101433\_contig\_number\_10502SRS104311\_contig\_number\_36267SRS1055034\_contig\_number\_9007SRS143780\_contig\_number\_contig-100\_2160.188927

2049040
52
species

SRS014412\_contig\_number\_13245SRS014979\_contig\_number\_31143SRS015578\_contig\_number\_352SRS015579\_contig\_number\_14888SRS015854\_contig\_number\_3536SRS016495\_contig\_number\_contig-100\_13052.86370SRS017821\_contig\_number\_26507SRS017859\_contig\_number\_contig-100\_386.55997SRS019161\_contig\_number\_4362SRS019381\_contig\_number\_contig-100\_1537.136460SRS019445\_contig\_number\_8348SRS019787\_contig\_number\_9229SRS020869\_contig\_number\_38239SRS043841\_contig\_number\_contig-100\_112.79577SRS044535\_contig\_number\_11766SRS046502\_contig\_number\_12669SRS047014\_contig\_number\_29778SRS049773\_contig\_number\_11725SRS050925\_contig\_number\_26306SRS050941\_contig\_number\_3073SRS051882\_contig\_number\_21075SRS052027\_contig\_number\_6017SRS053214\_contig\_number\_10552SRS058723\_contig\_number\_7535SRS063518\_contig\_number\_10860SRS065504\_contig\_number\_31791SRS075716\_contig\_number\_1253SRS075821\_contig\_number\_5474SRS076756\_contig\_number\_2807SRS077730\_contig\_number\_16510SRS078242\_contig\_number\_28796SRS078419\_contig\_number\_contig-100\_2518.175480SRS097889\_contig\_number\_41308SRS098571\_contig\_number\_57463SRS098827\_contig\_number\_contig-100\_6294.65002SRS098881\_contig\_number\_6521SRS1041036\_contig\_number\_1014SRS104485\_contig\_number\_2471SRS105153\_contig\_number\_6027SRS1055049\_contig\_number\_16351SRS1055083\_contig\_number\_contig-100\_1519.78222SRS142503\_contig\_number\_1602SRS142542\_contig\_number\_17085SRS142712\_contig\_number\_contig-100\_35.35SRS143417\_contig\_number\_15754SRS143722\_contig\_number\_14809SRS144183\_contig\_number\_29009SRS148253\_contig\_number\_8664SRS148424\_contig\_number\_32011SRS148721\_contig\_number\_25420SRS149784\_contig\_number\_24526SRS971275\_contig\_number\_20710

genus
1

SRS021484\_contig\_number\_contig-100\_6346.47117
33042

541000
1
family

1898205

SRS013951\_contig\_number\_17940
1
species


SRS014613\_contig\_number\_20419
1
species
1263000
